# The profile of hematinic deficiencies in patients with oral lichen planus: a case-control study

**DOI:** 10.1186/s12903-020-01229-w

**Published:** 2020-09-10

**Authors:** Zhe-xuan Bao, Xiao-wen Yang, Jing Shi, Yu-feng Wang

**Affiliations:** 1 Department of Oral Medicine, Shanxi Provincial People’s Hospital, Taiyuan, Shanxi China; 2 Department of Hospital Infection Control, Shanxi Provincial People’s Hospital: China, Taiyuan, Shanxi China; 3 Department of Oral Medicine, Shanghai Ninth People’s Hospital, College of Stomatology, Shanghai Jiao Tong University School of Medicine, 639 Zhizaoju Road, Shanghai, 200011 China

**Keywords:** Oral lichen planus, Hematinic deficiencies, Iron, Folate, Vitamin B12, Correlation

## Abstract

**Background:**

Oral lichen planus (OLP) is a relatively common mucocutaneous disorder, and its causative factors and pathogenesis are not fully understood. Existing studies on the association between hematinic deficiencies and OLP are limited and inconsistent. The aim of this study was to assess the hematinic deficiencies in a cohort of OLP patients and evaluate the correlation between hematinic deficiencies and OLP.

**Methods:**

A total of 236 OLP patients and 226 age-and-gender-matched healthy controls were enrolled in this study. The levels of hemoglobin (Hb), serum folate, vitamin B12 and ferritin were measured and compared between OLP patients and healthy controls. An REU (reticular/hyperkeratotic, erosive/erythematous, ulcerative) scoring system was adopted and compared between the OLP patients with and without hematinic deficiencies. The correlation between hematinic deficiencies and OLP was analyzed.

**Results:**

The frequencies of serum ferritin and vitamin B12 deficiency in OLP patients were both significantly higher than those of the healthy controls. According to gender and age, the profiles of hematinic deficiencies in OLP patients were significantly different. As for the REU score, no significant difference existed between OLP patients with and without hematinic deficiencies. Both serum ferritin deficiency and serum vitamin B12 deficiency were significantly correlated with OLP.

**Conclusions:**

The present study suggested a significant association between hematinic deficiencies and OLP. Iron, folate, and vitamin B12 levels in OLP patients should be monitored routinely. Further studies are warranted to explore the interactions between OLP and hematinic deficiencies.

## Background

Oral lichen planus (OLP) is a relatively common mucocutaneous disorder that affects 1 to 2% of the population, including mainly middle-aged adults, with a slight female predilection [1, 2]. It is widely accepted that OLP is an immune-related disorder, in which aberrant T-lymphocytes might play a vital role in its pathogenesis [1, 3]. Although abundant studies have been carried out, the exact causative factors and underlying pathogenesis of OLP are not fully understood and require further investigation [1–3].

Iron, folate and vitamin B12 deficiency are the most common hematinic deficiencies, which have been closely associated with some common oral mucosal diseases, such as recurrent aphthous stomatitis (RAS) and atrophic glossitis (AG) [4, 5]. In addition to affecting the integrity of the oral mucosa and increasing the risk of secondary infection in the oral cavity, hematinic deficiencies can also cause aberrant immune responses or contribute to psychological disorders, which may be involved in the pathogenesis of OLP [6–11]. However, existing studies on the association between hematinic deficiencies and OLP have been lacking and the conclusions were inconsistent [12–15]. Wu et al. proposed that OLP was one of the leading oral manifestations of iron deficiency anemia (IDA) and could be diagnosed in a third of patients with IDA in their oral mucosal exam [16]. In another study, the same team reported that the frequencies of serum vitamin B12, iron deficiencies and anemia in OLP patients were significantly greater than those in controls [15]. In contrast, an earlier study showed the comparable frequencies of hematinic deficiencies and anemia between OLP patients and healthy controls, suggesting that hematinic deficiencies may not be a predisposing factor for OLP [13]. Therefore, further determination on the association between hematinic deficiencies and OLP are required, which might provide the foundation for nutritional supplements in treating OLP and also give new insight into the possible interaction between hematinic deficiencies and OLP.

The current study was undertaken to assess the hematinic deficiencies in a cohort of OLP patients and evaluate the association between hematinic deficiencies and OLP. Furthermore, whether the severity of OLP is related to hematinic deficiencies was also analyzed.

## Methods

A total of 236 OLP patients (41 males and 195 females, mean age, 51.70 ± 13.99 years) and 226 gender- and age-matched healthy controls (52 males and 174 females, mean age, 49.46 ± 17.26 years) were enrolled in this retrospective study (Table 1). All patients were diagnosed consecutively from July 2015 to March 2019 at the Department of Oral Medicine, Shanxi Provincial People’s Hospital, China. Healthy controls were recruited from December 2014 to August 2017 at the same hospital. Sample size estimation was performed using two independent groups for binary data; the sample size formula was provided by Machin et al. [17]. Sample size calculation was based on the 2% prevalence of OLP, and the odds ratio (OR) was 5.0. Alpha was set as 0.05; the value of beta was considered as 0.1 in the formula. The required sample size for the OLP group and control group was at least 221, respectively. Hence, this study fulfilled the sample size requirements for further data analysis.

**Table 1:** Demographic data and the serum levels of vitamin B12, folate, ferritin, hemoglobin of the participants

	OLP (*n* = 236)	Health controls (*n* = 226)	*P*
Age ^a^	51.70 ± 13.99	49.46 ± 17.26	0.127
Male-to-female ratio ^b^	41:195	52:174	0.131
vitamin B12 (ng/L) ^c^	261.00 ± 171.75	306.50 ± 220.5	< 0.001
folate (ng/ml) ^c^	9.38 ± 5.90	11.50 ± 30.04	< 0.001
Ferritin (ng/ml) ^c^			
Male	126.80 ± 94.85	120.30 ± 130.38	0.629 ^c^
Female	54.20 ± 101.8	38.65 ± 65.62	0.458 ^c^
Hemoglobin (g/dL) ^c^			
Male	160.00 ± 18.50	156.00 ± 16.25	0.798 ^c^
Female	136.00 ± 16.00	136.00 ± 14.25	0.845 ^c^

*SD* Standard deviation; *QR* Quartile Range

^a^Independent-sample t test; Mean ± SD

^b^Chi-square test

^c^Wilcoxon’s rank-sum test, Median ± QR.

The diagnosis of OLP was established according to the recommended diagnostic criteria [3]. The characteristic clinical manifestations alone, such as bilateral grayish-white Wickham striae or papules, may allow diagnosis, especially if classic skin or other extraoral lesions exist concomitantly. Besides the clinical examination, a biopsy for histopathological examination was routinely performed. When necessary, direct immunofluorescence (DIF) was employed to differentiate from autoimmune blistering diseases, such as pemphigus and pemphigoid. Specifically, any patient suspected of having oral lichenoid lesions, including contact hypersensitivity reactions, drug-induced reactions, paraneoplastic pemphigus and chronic graft-vs-host disease, was not enrolled in the present study. A thorough medical history was obtained from each participant, and a standard clinical examination of the oral cavity was also performed. The duration of OLP, which was estimated from the first notice of oral discomfort, was recorded for each patient. All patients who were histologically confirmed to have epithelial dysplasia or diagnosed as having other oral mucosal lesions, such as leukoplakia, mucosal hyperkeratosis, erythema multiforme or discoid lupus erythematosus, were also excluded. In addition, patients having concomitant systemic disease, including benign or malignant tumors, HIV, rheumatic or autoimmune disease, uncontrolled thyroid disease, liver or kidney disease, gastrointestinal disease or a history of alcoholism were equally excluded from the study. None of the healthy controls exhibited any oral mucosal lesions or had relevant systemic disease. None of the participants had taken any nutritional supplements, including (but not limited to) folate, vitamin B12 or iron supplements, at least 3 months before the study.

This study was conducted in accordance with the Helsinki Declaration and approved by the ethics committee of Shanxi Provincial People’s Hospital (No. 20190302), and informed consent was obtained from each of the participants.

### Laboratory methods

After 12 h of overnight fasting, blood samples were obtained from all participants. The serum was separated and tested immediately. After internal quality control, the serum levels of folate, vitamin B12 and ferritin were measured by the chemiluminescence method (Beckman Coulter, DXI-800-Immunoassay System) in the Clinical Laboratory of Shanxi Provincial People’s Hospital. The accepted normal serum folate level and serum vitamin B12 level was 4.0–18.7 ng/mL and 180–914 ng/L, respectively. Serum ferritin level was used to assess iron status with a normal level of 11.0–306.8 ng/mL for females and 15–336.2 ng/mL for males. Serum vitamin B12, folate and ferritin deficiencies were defined as a serum level below their lower cutoff values, respectively. The hemoglobin (Hb) level was also determined and anemia was diagnosed when the Hb level was lower than the lower cut-off value (male, **<** 13 g/dL; female, **<** 12 g/dL).

### Clinical examination and scoring

The REU (reticular/hyperkeratotic, erosive/erythematous, ulcerative) scoring system was adopted to evaluate the severity of OLP as previously reported [13, 14]. Briefly, the oral cavity of each OLP patient was divided into 10 sites and the severity of the mucosal lesions in each site was scored according to the presence of reticular/hyperkeratotic, erosive/erythematous, and ulcerative lesions. Here, the REU score of each OLP patient was determined by the same well-trained specialist (ZX Bao).

### Data analysis

Statistical analyses were performed with SPSS software, version 22.0 (SPSS Inc., Chicago, Ill). The independent-samples *t* test was used to compare age between OLP patients and healthy controls. The mean serum levels of vitamin B12, folate, ferritin, and hemoglobin were compared using Wilcoxon’s rank-sum test. The male-to-female ratio, the frequencies of hematinic deficiencies and anemia between OLP patients and healthy controls were compared using the chi-square test. When the observed frequency was less than 1, Fisher’s exact test was applied. The Wilcoxon-Mann-Whitney rank sum test (U test) was used to statistically compare REU scores between the OLP patients with and without hematinic deficiencies. Logistic regression analysis was conducted to assess whether the age and gender were significant factors related to the presence of hematinic deficiencies (ferritin, folate and vitamin B12 deficiency) in the OLP patients and healthy controls, respectively. Spearman’s correlation coefficient was applied to evaluate the association between OLP and hematinic deficiencies. A *P*-value < 0.05 was accepted as statistically significant.

## Results

1. Serum levels of vitamin B12, folate, ferritin, hemoglobin in OLP patients and healthy controls

Compared with healthy controls, OLP patients had significantly lower serum levels of vitamin B12 and folate (both *P* < 0.001, Table 1). For both males and females, the levels of ferritin and hemoglobin were not significantly different between the two groups (Table 1).

2. Hematinic deficiencies in OLP patients compared with healthy controls

Here, the frequency of overall hematinic deficiencies means the frequency of the subjects with at least one deficiency in serum folate, ferritin and vitamin B12. The frequency of overall hematinic deficiencies was 43.64% (103/236) in the OLP patients vs 12.39% (28/226) in the healthy controls. There was a statistically significant difference between the two groups (*P* < 0.001, Table 2). Similarly, the frequencies of serum ferritin deficiency and serum vitamin B12 deficiency in OLP patients were both significantly higher than those of the healthy controls (both *P* < 0.001, Table 2). However, no statistically significant difference was found in the frequency of serum folate deficiency between the OLP patients and healthy controls (4.24% *vs*1.33%, *P* = 0.059). Compared with the health controls, anemia was much more common in OLP patients (9.75% vs 3.98%, *P* = 0.015).

3. Hematinic deficiencies in OLP patients according to gender and age

**Table 2:** Hematinic deficiencies in the OLP patients compared with the healthy controls

	OLP patients	Healthy controls	χ^2^	*p*
Overall hematinic deficiencies	43.64% (103/236)	12.39% (28/226)	52.366	< 0.001
Serum ferritin deficiency	21.19%(50/236)	7.52% (17/226)	16.147	< 0.001
Serum folate deficiency	4.24% (10/236)	1.33% (3/226)	3.574	0.059
Serum vitamin B12 deficiency	27.54% (65/236)	7.52% (17/226)	31.696	< 0.001
Anemia	9.75% (23/236)	3.98% (9/226)	5.949	0.015

Compared with the male OLP patients, the female OLP patients had a significantly higher frequency of serum ferritin deficiency (4.88% vs 24.62%, *P* = 0.005, Table 3). On the contrary, serum folate deficiency was more common in male OLP patients (*P* < 0.001). No significant differences in serum vitamin B12 deficiency or anemia were found between male and female OLP patients (*P* = 0.511 and *P* = 0.148, respectively).

**Table 3:** The statistical analysis of hematinic deficiencies in OLP patients according to gender and age

Hematinic deficiencies	OLP patients								
	Gender				Age				
	Male	Female	χ^2^	*P*	≤40	41–60	≥61	χ^2^	*P*
Serum ferritin deficiency	4.88% (2/41)	24.62% (48/195)	7.904	0.005	29.16% (14/48)	28.23% (35/124)	1.56% (1/64)	20.271	< 0.001
Serum folate deficiency	17.07% (7/41)	1.54% (3/195)	16.501	< 0.001	8.33% (4/48)	2.42% (3/124)	4.68% (3/64)	3.027	0.220
Serum vitamin B12 deficiency	31.71% (13/41)	26.67% (52/195)	0.431	0.511	22.92% (11/48)	31.45% (39/124)	23.44% (15/64)	2.005	0.367
Anemia	2.44% (1/41)	11.28% (22/195)	2.090	0.148	10.42% (5/48)	13.71% (17/124)	1.56% (1/64)	7.112	0.029

The frequencies of hematinic deficiencies were also compared among age subgroups of the OLP patients. Significant differences in serum ferritin deficiency and anemia were revealed among age subgroups of OLP patients (*P* < 0.001, *P* = 0.029, respectively, Table 3). However, there was no significant difference among age subgroups in either serum folate or vitamin B12 deficiency (*P* = 0.220 and *P* = 0.367, respectively).

4. The correlation between two factors (age and gender) and presence of hematinic deficiencies in OLP patients

Both age and gender were significantly correlated with the presence of serum ferritin deficiency in the OLP patients (Table 4). However, neither age nor gender was significantly correlated with serum vitamin B12 deficiency in the OLP patients (*P* = 0.413 and *P* = 0.623, respectively, Table 4). Gender was significantly associated with serum folate deficiency in OLP patients (*P* < 0.001). Such significant correlation was also observed between age and anemia (*P* = 0.021, Table 4). For comparison, the same logistic regression analysis was conducted in the healthy controls and no significant correlation was found between the two factors and the presence of hematinic deficiencies (Table 4).

5. Analysis of disease duration in OLP patients

**Table 4:** Logistic regression analysis on the correlation between the two factors (age and gender) and the presence of hematinic deficiencies

	Factor	OLP patients				Healthy controls			
		*β*	OR	95%CI	*P*	*β*	OR	95% CI	*P*
Serum ferritin deficiency	Age	0.074	1.077	1.045–1.109	< 0.001	0.029	1.029	0.998–1.061	0.066
	Gender	−2.798	0.061	0.012–0.300	0.001	−18.763	–	–	0.997
Serum folate deficiency	Age	0.002	1.002	0.962–1.043	0.933	0.057	1.059	0.986–1.136	0.115
	Gender	2.567	13.028	3.130–54.222	< 0.001	2.437	11.435	0.928–140.956	0.057
Serum vitamin B12 deficiency	Age	0.009	1.009	0.988–1.030	0.413	−0.003	0.997	0.968–1.027	0.866
	Gender	0.187	1.205	0.572–2.541	0.623	0.339	1.403	0.456–4.322	0.555
Anemia	Age	0.040	1.041	1.006–1.077	0.021	0.015	1.015	0.976–1.056	0.460
	Gender	−2.010	0.134	0.017–1.079	0.059	−18.155	–	–	0.997

In this study, 63.56% of OLP patients (150/236) had not experienced oral symptoms more than 6 months. Among these patients, serum folate deficiency was detected in six patients, serum vitamin B12 deficiency in 44 patients and serum ferritin deficiency in 23 patients. A shorter duration, 1 month or less, was found in 30 OLP patients. Among these, seven patients, five patients and no patient had serum vitamin B12 deficiency, serum ferritin deficiency or serum folate deficiency, respectively.

6. Comparison of REU scores between OLP patients with and without hematinic deficiencies

The REU score (median ± IQR) of the OLP patients with one or more (two or three) deficiencies in serum folate, ferritin and vitamin B12 was 5 ± 3. For OLP patients without any deficiency, the REU score was 5 ± 3.75. The rank sum test revealed no significant difference between them (*P* = 0.824, Table 5). Separate analysis also showed that no significant difference existed between OLP patients with and without serum ferritin deficiency (*P* = 0.783), folate deficiency (*P* = 0.173), vitamin B12 deficiency (*P* = 0.493) or anemia (*P* = 0.723, Table 5).

7. Statistical analysis of the association between hematinic deficiencies and OLP.

**Table 5:** The comparison of REU scores between the OLP patients with and without hematinic deficiencies

OLP patients		REU scores (median ± IQR)	*P*
Overall hematinic deficiencies	with	5 ± 3	0.824
	without	5 ± 3.75	
Serum ferritin deficiency	with	5.25 ± 3.5	0.783
	without	5 ± 3	
Serum folate deficiency	with	4.5 ± 1.75	0.173
	without	5 ± 3.5	
Serum vitamin B12 deficiency	with	5 ± 3.25	0.493
	without	4.5 ± 3	
Anemia	with	6 ± 4	0.723
	without	5 ± 3	

*REU* (reticular/hyperkeratotic, erosive/erythematous, ulcerative), *IQR* (Interquartile range).

Spearman’s correlation coefficient revealed that both serum ferritin deficiency and serum vitamin B12 deficiency were significantly correlated with OLP (*R* = − 0.189, *P* **<** 0.001; *R* = − 0.262, *P* < 0.001, respectively). A borderline, but not statistically significant correlation was observed between serum folate deficiency and OLP (*R* = − 0.088, *P* = 0.059). Moreover, anemia was not significantly associated with OLP (*R* = − 0.05, *P* = 0.284).

## Discussion

Consistent with previous studies [15, 18, 19], the present study exhibited a significantly higher frequency of hematinic deficiencies in OLP patients than in healthy controls. Moreover, a significant association between hematinic deficiencies and OLP was also demonstrated. However, this association should be interpreted with caution. Since OLP is a chronic condition with periods of exacerbation and remission, it may cause discomfort or pain and difficulty in eating and drinking [20–22]. A study on dietary changes in 48 patients with oral vesiculoulcerative diseases, of whom most were diagnosed with OLP, showed that even patients with mild forms of the disease would change their eating habits for extended periods, which might negatively affect the patients’ nutritional status [22]. From this point of view, it seems reasonable to conclude that the hematinic deficiencies might be the result of OLP. Nevertheless, it should be noted that the occurrence of hematinic deficiencies usually takes several months to years to appear [8, 9]. For example, due to the important hepatic stores and enterohepatic circulation, vitamin B12 deficiency would occur only if the daily intake has been insufficient for years [8]. In this study, we found that hematinic deficiencies had already existed in many of the enrolled patients, which were unlikely caused by OLP because of their rather short duration. Hence, we could speculate that, at least for some OLP patients, hematinic deficiencies occur earlier than the onset of OLP.

On the other hand, there is yet no direct evidence suggesting that hematinic deficiencies are involved in the pathogenesis of OLP, but several plausible mechanisms are proposed and need to be investigated in further studies. First, inadequate iron, folate or vitamin B-2 can significantly alter the immune response and affect cell-mediated immunity [10, 11, 23, 24]. For example, significant suppressed natural killer (NK) cell activity was noted in patients with vitamin B12 deficiency compared with control subjects and the decreased activity could be restored after vitamin B12 supplementation [25]. Coincidentally, significantly decreased NK cell activity was also found in LP patients [26, 27]. Second, the levels of vascular cell adhesion molecule-1 (VCAM-1), an inducible adhesion protein in endothelial cells, were significantly higher in patients with IDA compared with controls [28]. The expression of VCAM-1 in OLP was equally significantly increased [29]. Third, it has been demonstrated that psychological disorders, such as anxiety and depression, might play an important role as a trigger for OLP and might also be responsible for many relapses [6]. Notably, vitamin B12 deficiency or insufficiency might contribute to the etiopathogenesis of depression. Folic acid and vitamin B12 supplements were recommended for inclusion in treating depression [30]. However, the question remains to be elucidated since more than half of OLP patients did not have hematinic deficiencies in the present study. Prospective studies on the incidence of OLP in patients having hematinic deficiencies with large-sample and long-term follow-up are needed to provide more clinical evidence.

The REU scoring system is a semiquantitative method with less subjectivity and good reproducibility and has been validated to be much more accurate for comparing the severity of oral lichenoid lesions [31–33]. In a previous study, the inter- and intra-observer agreement of this scoring system was assessed with the finding that the Spearman correlation coefficient was 1.0 and 0.98, respectively [31]. To the best of our knowledge, this is the first report of using a disease scoring system (DSS) in investigating the association between the severity of OLP and hematinic deficiencies. In this study, no significant difference in REU scores was found between OLP patients with and without hematinic deficiencies, suggesting that the hematinic deficiencies may not directly correlate with the severity of OLP. This finding might be due to the well-recognized phenomenon that the clinical character of OLP can alleviate or aggravate even in a short time, especially when there are local irritations and trauma in the oral cavity [2, 3]. However, the levels of serum ferritin, folate and vitamin B12 in the human body could not fluctuate so rapidly. The biomarkers that can sensitively reflect the severity of OLP still warrant further investigation.

One limitation of the present study is that there are still no “gold standard” laboratory tests for hematinic deficiencies in routine clinical practice [34, 35]. The sensitivity and specificity of available assays still need to be improved [34, 35]. Some newer assays, such as measuring holotranscobalamin II (holoTC), and additional tests of serum total homocysteine (tHcy), methylmalonic acid (MMA), transferrin receptor and red blood cell folate (RBC folate) are recommended for further studies [35].

Based on our findings, hematological screening for hematinic deficiencies should be included in routine laboratory examination of OLP patients. Several studies have proposed that vitamin replacement may improve the general health of OLP patients and increase their healing ability [12, 36]. Therefore, compensation of hematinic deficiencies with adequate nutritional supplements or in combination with other drugs, is supposed to produce improved therapeutic effects on OLP patients [12, 36]. In addition, dietary assessment and guidance to maintain adequate nutrition and optimal quality of life should be considered as a component of OLP management [20, 22].

## Conclusions

In conclusion, the present study suggests a significant association between hematinic deficiencies and OLP. Folate, vitamin B12 and iron levels in OLP patients should be monitored routinely. Further studies are warranted to explore the interactions between OLP and hematinic deficiencies.

## Data Availability

The datasets used and analyzed during the current study are available from the corresponding author on reasonable request.

## Abbreviations

AG: Atrophic glossitis
DIF: Direct immunofluorescence;
DSS: Disease scoring system
Hb: Hemoglobin
IDA: Iron deficiency anemia
OLP: Oral lichen planus
RAS: Recurrent aphthous stomatitis
REU: Reticular/hyperkeratotic, erosive/erythematous, ulcerative.
